# Bacterial Resistance to Antimicrobial Agents

**DOI:** 10.3390/antibiotics10050593

**Published:** 2021-05-17

**Authors:** Manuel F. Varela, Jerusha Stephen, Manjusha Lekshmi, Manisha Ojha, Nicholas Wenzel, Leslie M. Sanford, Alberto J. Hernandez, Ammini Parvathi, Sanath H. Kumar

**Affiliations:** 1 Department of Biology, Eastern New Mexico University, Portales, NM 88130, USA; Manisha.Ojha@enmu.edu (M.O.); Nicholas.Wenzel@enmu.edu (N.W.); Leslie.Sanford@enmu.edu (L.M.S.); Alberto.J.Hernandez@enmu.edu (A.J.H.); 2 Post-Harvest Technology, ICAR-Central Institute of Fisheries Education, Seven Bungalows, Andheri (W), Mumbai 400061, India; jerusha.phtpa702@cife.edu.in (J.S.); manjusha@cife.edu.in (M.L.); sanathkumar@cife.edu.in (S.H.K.); 3 CSIR-National Institute of Oceanography, Regional Centre, Kochi 682018, India; parvathi@nio.org

**Keywords:** bacteria, antimicrobial resistance, infection, pathogenesis, multidrug resistance

## Abstract

Bacterial pathogens as causative agents of infection constitute an alarming concern in the public health sector. In particular, bacteria with resistance to multiple antimicrobial agents can confound chemotherapeutic efficacy towards infectious diseases. Multidrug-resistant bacteria harbor various molecular and cellular mechanisms for antimicrobial resistance. These antimicrobial resistance mechanisms include active antimicrobial efflux, reduced drug entry into cells of pathogens, enzymatic metabolism of antimicrobial agents to inactive products, biofilm formation, altered drug targets, and protection of antimicrobial targets. These microbial systems represent suitable focuses for investigation to establish the means for their circumvention and to reestablish therapeutic effectiveness. This review briefly summarizes the various antimicrobial resistance mechanisms that are harbored within infectious bacteria.

## 1. Introduction

Bacteria as microbial pathogens are causative agents of life-threatening infectious diseases [1]. Such pathogenic bacteria produce alarming numbers in terms of morbidity and mortality outcomes [2,3]. One crucial avenue towards bacterial pathogenesis involves the reduction in the therapeutic effects of antibacterial chemotherapy [4,5]. Throughout their evolutionary history, bacterial pathogens have developed various means of resisting the inhibitory and bactericidal consequences of antimicrobial agents [4]. Such antimicrobial resistance systems involve the engagement of bacterial molecular and cellular-based machinery [6]. Interestingly, the selection of a bacterial variant with resistance to a single antimicrobial agent frequently manifests the emergence of a multidrug resistance characteristic in the new mutant [7]. Newly emerged bacterial pathogens with resistance to multiple antibacterial agents can result in compromised efficacy in the treatment of infection [3,8].

**Figure 1:**
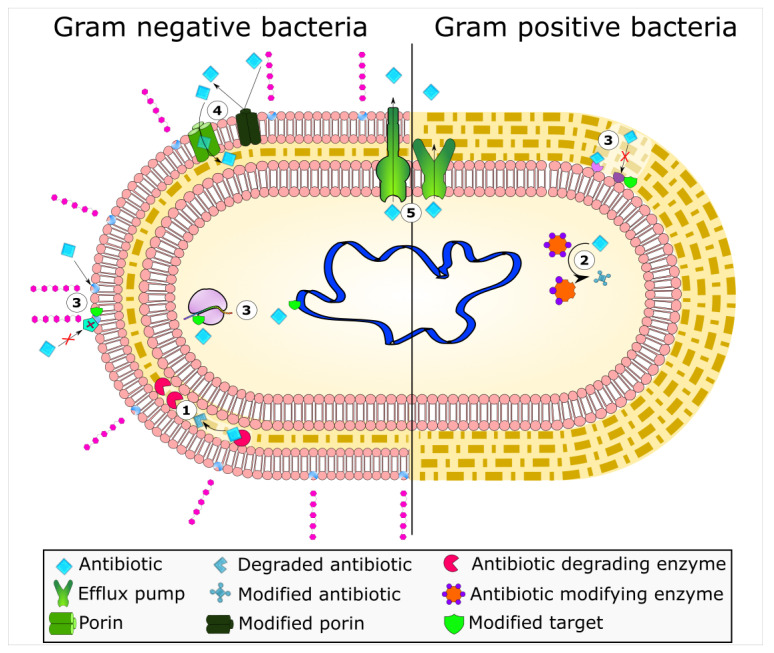
Bacterial mechanisms of resistance to antimicrobial agents. The common mechanisms of antibiotic resistance in bacteria are enzymatic hydrolysis (**1**), enzymatic modifications of antibiotics by group transfer and redox process (**2**), modifications of antibiotic targets (**3**), reduced permeability to antibiotics by modifications of porins (**4**), and active extrusion of antibiotics by membrane efflux pumps (**5**).

Mechanisms of antimicrobial resistance include the active export systems within the membranes of bacteria, prevention of antimicrobial entrance into cells of pathogenic bacteria, enzymatic destruction of antimicrobial agents, production of thick biofilms, modified targets of antimicrobials, and bacterial sites of action that are protected from antimicrobials, (Figure 1) [2,4]. Furthermore, multidrug-resistant bacteria have developed mechanisms that confer the DNA transfer of genetic determinants of resistance to pathogenic species in the clinical setting, the food production industry, the human gut, and in agriculture [9].

Thus, new strategies for the circumvention of bacterial resistance to antimicrobial agents are desired [10]. In order to discover novel approaches to address multiple antimicrobial resistances in these microbial pathogens, however, it is necessary to attain a clear understanding of these resistance systems at the molecular and cellular levels. For young and new investigators, here, we consider an introductory overview of each of these disparate bacterial resistance mechanisms here.

## 2. Enzyme-Based Antimicrobial-Inactivation Systems

Along the timeline of antibiotic discovery and introduction, several enzymatic mechanisms of antibiotic inactivation were also discovered. Although very few novel mechanisms of antibiotic resistance have been reported in recent times, several new variants of known enzymes that endow bacteria with resistance to newly introduced drugs have emerged, suggesting that the bacterial response to new antibiotics or the modified versions of existing antibiotics is swift. The enzymatic mechanisms of antibiotic resistance include hydrolysis, group transfer, and redox processes [4]. In terms of diversity, evolution, and spread, antibiotic resistance enzymes contribute remarkably to the bacterial ability to overcome antibiotic pressure. The β-lactamases are the oldest known and the most diverse antibiotic degrading enzymes that cleave the β-lactam ring of the penicillin group of antibiotics and render them ineffective. The first such β-lactamase was discovered soon after the first antibiotic penicillin was in clinical use. Scientific evidence suggests the existence of β-lactamases before penicillin was clinically employed, emphasizing that the production of antimicrobial compounds and the mechanisms to endure them occur in parallel in the environment [11]. Bacteria that produce antibiotics apparently require mechanisms to overcome the lethal effects of the compounds, and these are in the form of concurrent production of degradative enzymes, mutations in targets of antibiotics, or active extrusion of antibiotics from the cell so that the antibiotic-producing cell is protected. However, the selection pressure created due to the extensive use of antibiotics in humans and animals propagated the resistant clones of bacteria in clinical and food production environments. In due course of time, genetic exchange mechanisms facilitated the wider dissemination of resistance traits in bacterial communities. The introduction of more antibiotics, newer as well modified, augmented the process of evolution and spread of resistance mechanisms. Since the majority of the antibiotics introduced in the last two decades are mostly the modified versions of existing antibiotics belonging to the same classes (e.g., β-lactams), a few mutations in the enzymes could render bacteria quickly resistant to them [11].

The β-lactams constitute the largest group of clinically used antibiotics, comprising of penicillins, cephalosporins of different generations, monobactams, and carbapenems, all of which are characterized by the presence of 3-carbon, 1-nitrogen containing β-lactam ring. The β-lactam antibiotics inhibit the bacterial proteins known as penicillin-binding proteins (PBPs), which perform the critical role of peptide cross-linking during peptidoglycan cell wall biosynthesis. The structural mimicry of the d-Ala-d-Ala terminal fragment of cross-linking peptide by β-lactams facilitates competitive inhibition of PBPs [12], which stops the cell wall synthesis leading to bacterial cell lysis and death [13]. However, bacteria gain resistance to lactam antibiotics by modifying their PBPs, which are no longer susceptible to binding by the antibiotic. Alternatively, bacteria produce powerful lactamases that degrade antibiotics before they can bind with the PBPs. Since their discovery in the early 1940s, the family of β-lactamases has grown seamlessly, with more than 300 enzymes identified globally [14,15].

**Figure 2:**
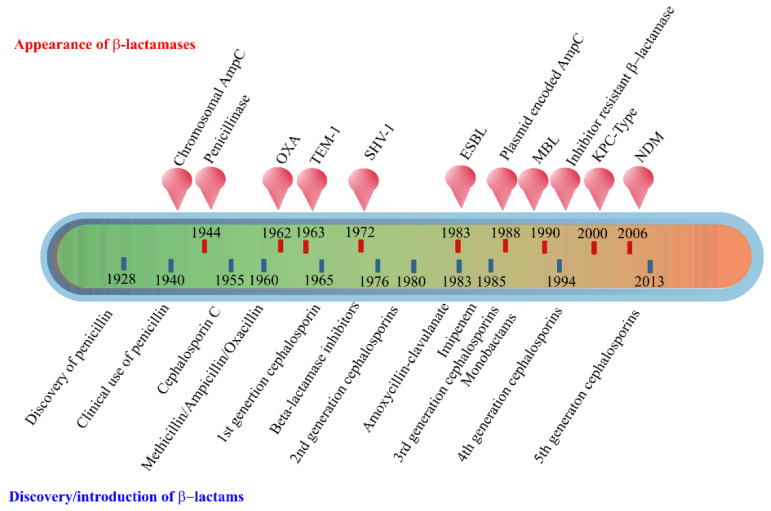
Evolution of β-lactamases. Within five decades of discovering the first penicillin-degrading enzyme, β-lactamases capable of hydrolyzing most β-lactam antibiotics, and resistance to inhibitors have emerged. The ability to tolerate a broad spectrum of β-lactams and inhibitor combinations is bolstered by the presence of multiple β-lactamase-encoding genes in a single pathogen.

The early β-lactamases were penicillinase enzymes that degraded penicillin, which started appearing rapidly in clinical bacteria [16,17]. The introduction of modified, semisynthetic penicillins such as methicillin, ampicillin, and amoxicillin resulted in the gradual appearance of β-lactamases capable of degrading them. The first plasmid-borne transferrable β-lactamase was TEM-1, followed by TEM-2 and SHV-1 enzymes [18,19]. TEM is the most common mechanism of ampicillin resistance compared to less prevalent SHV-1, although both have the same affinity for this antibiotic. TEM and SHV share 60% amino acid similarity between them and are inhibited by clavulanic acid, tazobactam, and sulbactam. The discovery of cephalosporin C in the early 1960s heralded an era of synthetic cephalosporins, which was thought to fend off β-lactamases. Structurally, cephalosporins have their β-lactam ring fused to a six-membered dihydrothiazine ring compared to penicillins in which the β-lactam is fused with a five-membered thiazolidine ring [20]. Subsequently, carbapenem and monobactam groups of β-lactam antibiotics with structurally variant lactam rings were discovered from natural sources and formed the basis for the synthesis of similar compounds with modifications. However, the enzymes extended-spectrum β-lactamases (ESBLs) that could hydrolyze a wide range of cephalosporins emerged from TEM and SHV lactamases by point mutations [18]. ESBLs hydrolyze a broad spectrum of cephalosporins, including first, second, third-generation cephalosporins and aztreonam, but not cephamycins and carbapenems, and are inhibited by clavulanic acid [18,21]. As a consequence of mutations and the expansion of the substrate range, ESBLs have a lesser affinity for classical β-lactams compared to their ancestral β-lactamases. Subsequently, CTX-M type ESBLs with high affinity for cefotaxime emerged independent of TEM and SHV lactamases, and these supposedly evolved from β-lactamases of *Kluyvera* spp. [22]. Over the years, CTX-M has overtaken other ESBLs in terms of number and global distribution, with more than 230 types identified to date. Figure 2 shows the timeline of the evolution of β-lactamases in relation to the introduction of β-lactam antibiotics for clinical use.

The initial efforts to classify β-lactamases were based on their functional characteristics such as the substrate-inhibitor profiles, protein molecular weight, isoelectric point, etc. [12,14,23]. A second approach employed amino acid sequence similarities and enzymatic activities to classify β-lactamases into four main groups, of which groups A, C, and D are serine β-lactamases, while class B is composed of metallo β-lactamases that require active site zinc ion(s) for their hydrolytic activities [12,24]. Group A enzymes form the largest group of lactamases comprising some of the critical resistance enzymes such as TEM, SHV, and CTX-M type of β-lactamases. Other important ESBLs include the carbapenem hydrolyzing KPC type ESBLs originally reported from *Klebsiella pneumoniae*, which have an expanded substrate spectrum encompassing the cephalosporins and carbapenems but susceptible to inhibition by clavulanates and boronic acid [23,25]. The chromosomally encoded AmpC (class C) cephalosporinases described early in the timeline of discovery of β-lactamases have no homology with penicillinases and thus constitute a distinct group of enzymes [26,27]. Commonly found in Enterobacteriaceae, AmpC enzymes are inducible and are produced at low basal levels, and preferentially hydrolyze cephalosporins including cefoxitin but not cefepime. These are generally resistant to inhibition by clavulanic acid, sulbactam, or tazobactam. The metallo-β-lactamases or MBLs belonging to class B have vigorous hydrolytic activities against carbapenems and are also active against a range of cephalosporins [28,29]. In 2009, a new variant New Delhi Metallo-β-lactamase (NDM), emerged, and since then, it has been reported from all over the world [29]. NDM confers resistance to all β-lactam antibiotics except aztreonam, and the plasmid carrying *bla*_NDM_ gene harbors resistance markers for several other antibiotics. VIM and IMP are other important class B carbapenemases commonly encountered in Enterobacteriaceae.

The OXA type enzymes belonging to the Class D lactamase group were originally discovered as plasmid-encoded oxacillin hydrolyzing enzymes in lactose non-fermenting bacteria such as *Pseudomonas*, *Acinetobacter,* and *Shewanella*, and later in Enterobacteriaceae through plasmid exchange [30,31]. These enzymes are poorly inhibited by lactamase inhibitors such as clavulanic acid. Although OXA lactamases have a narrow substrate range composed of penicillins, cloxacillin, and oxacillin, the enzymes evolved to hydrolyze extended-spectrum cephalosporins and carbapenems through point mutations, and these abilities vary among different OXA types [28,32].

The β-lactamase mediated antimicrobial resistance is widespread among ESKAPE (*Enterococcus*, *S. aureus*, *K. pneumoniae*, *A. baumannii*, *P. aeruginosa,* and *E. coli*) group of organisms, infections with which are usually associated with a significantly higher economic burden and highest risk of mortalities [33,34]. The World Health Organization (WHO) has recognized carbapenem-resistant Enterobacteriaceae (CRE) as a serious global health scourge for which the development of new antimicrobials is critically needed [35].

Enzymatic hydrolysis is also a common mechanism of resistance against macrolides, rifampicin, and fosfomycin. Many Enterobacteriaceae members produce plasmid-encoded esterases EreA and EreB that hydrolyze the macrolactone ring of 14- and 15-membered macrolides such as erythromycin A, clarithromycin, and azithromycin [36,37]. The structurally altered macrolide antibiotic will no longer be able to bind to its preferred target site in the ribosome [38].

Another important mechanism of enzymatic degradation is associated with the manganese ion (Mn^2+^)-dependent, chromosomally-encoded FosX that uses water to cleave the epoxide ring of fosfomycin. Other fosfomycin modifying metalloenzymes include FosA, FosB, and two epoxide kinases FomA and FomB [39]. FosA is a Mn^2+^ and K^+^-dependent glutathione-S-transferase, while FosB is a Mg^2+^ thiol-S-transferase. The mechanism involves adding glutathione or thiol groups to the oxirane ring of fosfomycin resulting in an inactive drug [40]. FomA and FomB kinases utilize ATP and Mn^2+^ ions to phosphorylate the oxirane ring of fosfomycin [39].

Tetracyclines are in use for over 70 years as widely used antibiotics in human and animal medicine [41]. Tetracycline is broken down by a monooxygenase enzyme Tet(X), which is oxygen- and FAD-dependent [42]. Tet(X) monohydroxylates break down tetracyclines at position 11a, followed by non-enzymatic degradation. Similarly, enzymatic monoxygenation of the naphthyl group of rifamycin antibiotics by monooxygenases (Rox) inactivate them by leading to the linearization of the naphthoquinone or naphthohydroquinone ring [43].

Enzymatic modification of antibiotics by the transfer of functional groups, such as acyl, glycosyl, ribosyl, nucleotidyl, phosphoryl, or thiol groups, confers resistance to a range of antibiotics, including aminoglycosides, rifamycins, macrolides, epoxides, and chloramphenicol [44]. The aminoglycoside modifying enzymes (AME) responsible for resistance to different aminoglycoside antibiotics include *N*-acetyltransferases (AAC), *O*-adenyltransferases (ANT), and *O*-phosphotransferases (APH). These enzymes catalyze the modification of various hydroxyl or the amino groups of the aminoglycosides resulting in their inability to bind to their 30S ribosomal targets [45]. Similarly, in Gram-negative bacteria, a plasmid-encoded ADP-ribosyltransferase (Arr-2) is commonly responsible for rifampin resistance [46]. Similarly, chloramphenicol is modified by acetyl-CoA-dependent acetylation of its 3-hydroxyl group by chloramphenicol acetyltransferase (CAT) enzymes [47]. The modified antibiotic does not bind to its target site, the 50S subunit of ribosomes. CATs are widely distributed among Gram-positive and -negative bacteria and show little amino acid sequence similarities, with only 25 amino acid residues conserved among all CAT variants [47].

## 3. Alteration of Antimicrobial Targets

As bacterial enzymes mentioned above alter drug structures, the drug targets may likewise be altered, preventing drug binding and, thus, conferring resistance. Antimicrobial targets play vital roles in microbial growth or survival and, thus, serve as potentially useful targets for mitigating infection. In addition, these targets must differ or be completely absent from humans or the animal species being treated with an antimicrobial to allow for a selective mode of action. A classic example of such a target is peptidoglycan. Peptidoglycan is essential to the growth and survival of many bacterial species and has a chemical structure that is not present in the mammalian hosts they infect. This allows for the targeting of enzymes responsible for the synthesis and assembly of peptidoglycan. The function of proteins associated with these target sites makes it non-viable for a bacterium to evolve resistance by removing these proteins. However, mutations that allow for continued functionality while reducing the ability of an antimicrobial agent to bind them at the target site have been a veritable regularity in the arms race between antimicrobial substances and antimicrobial-resistant bacteria. In addition to peptidoglycan, alteration in target sites has been attributed to ribosomes, nucleic acid enzymes, and lipopolysaccharides [48].

**Figure 3:**
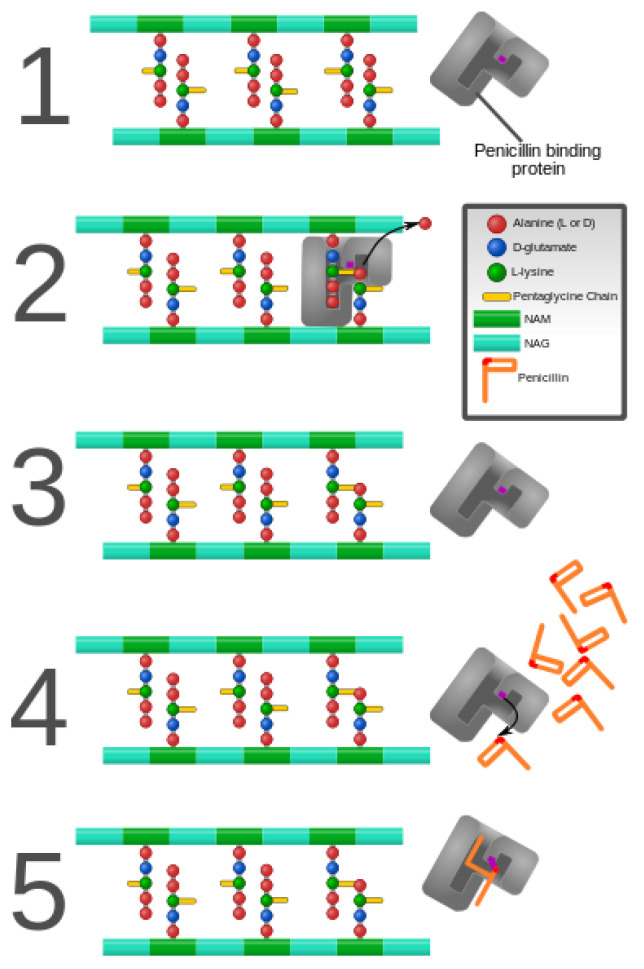
Penicillin and penicillin-binding protein of the bacterial cell wall. (**1**) The peptidoglycan layer of a bacterial cell wall harbors the repeating moieties of *N*-acetylglucosamine (NAG) and *N*-acetylmuramic acid (NAM). The NAM subunits bind short variable peptide chains, usually l-Ala and two distal d-Ala residues. (**2**) The PBP cross-links the peptide side chain, releasing a free Ala. (**3**) Upon cross-linking, the PBP dissociates from the cell wall. (**4**) Penicillin binds the PBP active site, affecting its enzyme activity. (**5**) The β-lactam ring of penicillin is cleaved during its reaction with PBP. Penicillin stays covalently bound PBP, permanently inhibiting the active site. Altered PBPs, such as PBP2a, are unable to accommodate penicillin-binding, preventing cell wall synthesis inhibition [48,49].

As discussed previously in this review, peptidoglycan inhibition by glycopeptides involves the binding of the peptidyl-d-alanyl-d-alanine terminus of peptidoglycan precursors. This binding prevents integration via the transglycosylase activity of these precursors into the cell wall [49], as shown in Figure 3.

PCBs are one mechanism for antimicrobial resistance, but the peptidoglycan precursors themselves can undergo alteration, which reduces the affinity of antimicrobials without the involvement of enzymatic inactivation. Such is the case with *Enterococcus faecium* and *E. faecalis,* which have been discussed in the literature as developing resistance by acquiring one of two related gene clusters encoding VanA and VanB [50,51]. These gene clusters produce a modified terminus that contains d-alanyl-d-lactate as opposed to d-alanyl-d-alanine [50]. This alteration leads to glycopeptides having a much lower binding affinity [52]. Thus, these gene clusters, found on transposable elements, have allowed the spread of modified targets in enterococci. Similarly, there are rarer but related gene clusters that have been shown to modify peptidoglycan precursors, such as those encoding VanD [53], VanE [54], and Van G [55].

Ribosomes, serving the vital role of protein synthesis, are common to both prokaryotic and eukaryotic organisms but differ quite vastly from one another in structure, making them another suitable candidate for antimicrobial targeting [56]. The 50S ribosomal unit serves as the binding site for macrolide, lincosamide, and streptogramin B [57]. Recalcitrance to these specific antimicrobials is known as MLS(B) type resistance [57], and it results from a post-transcriptional modification of the 23S rRNA component of the 50S ribosomal subunit that is involved with methylation or dimethylation of key adenine bases in the peptidyl transferase functional domain [58]. Mutations in the 23S rRNA, close to the site of methylation have also been associated with resistance to the macrolide group of antibiotics in a range of organisms, such as *Helicobacter pylori* [59] and propionibacteria [60]. Macrolide resistance in S. *pneumoniae* has been attributed to an alteration in the L4 and L22 proteins of the 50S subunit [61,62]. Oxazolidinones bind to the 50S subunit but have a more complex set of interactions associated with their mechanism of action [63]. The translocation of peptidyl-tRNA from the A site to the P site is hindered by this class of antibiotics, but enterococci have been documented to have an altered the P site through the substitution of U in place of G in the peptidyl transferase region (position 2576) of the 23S rRNA, thus resulting in a lowered binding affinity in the 50S subunit for this class of antibiotics [64,65,66]. Mutations more closely associated with the A site have been found in *E. coli* at positions 2032 and 2447 which confer resistance to the oxazolidinone drug linezolid [67].

The 30S ribosomal unit is the target of tetracycline and of aminoglycosides, which function by preventing the decoding of mRNA [68]. Mutations of the gene encoding 16S rRNA confer resistance to this class of antimicrobials [69]. Suzuki and colleagues discovered that substitutions at positions 1400, 1401, and 1483 led to kanamycin resistance in clinical isolates of *Mycobacterium*, and further strengthened the claim that these changes led to resistance by identifying their absence in kanamycin-sensitive *Mycobacterium* isolates [70]. Position 1400 was the most common substitution, featuring an A to G change [70]. The same A to G substitution at position 1408 led to high resistance against amikacin, kanamycin, gentamicin, tobramycin, and neomycin in clinical isolates of *Mycobacterium abscessus* [71].

## 4. Protection of Antimicrobial Targets

The previous section discussed antimicrobial resistance via the alteration of drug targets. However, targets may also be protected by other specific factors. One of the significant lines of defense against an antimicrobial is the bacterial cell wall [72]. Thought to have evolved initially for protection against the cell’s internal turgor pressure, this structure also acts as a physical barrier to encase the cytoplasm and cell membrane from the external world [72,73,74]. Prokaryotic cell walls are made up of linear glycan strands cross-linked by small peptides [75]. This peptidoglycan (murein) sacculus helps to limit which substances can continue on towards the cell membrane and ultimately into the cytoplasm [76]. Peptidoglycan also plays an essential role in bacterial growth and proliferation [77]. The crucial role that peptidoglycan and the cell wall play has caused most species of bacteria, except for *Mycoplasma* and l-form bacteria, to contain their structures [78]. While the cell wall helps protect cytoplasmic antimicrobial targets, it also ended up being the target for the first natural antibiotic, penicillin, which prevents the complete formation of this barrier by inhibiting peptide cross-linking to occur [79]. With a faulty protective structure, the cell becomes vulnerable to its internal environment and the external environment, leading to cell death [80].

With this mechanism of protection compromised due to the advent of β-lactam antibiotics, prokaryotes began to synthesize another tier of protection: β-lactamases [16]. These enzymes help to protect the peptidoglycan cell wall from β-lactam antibiotics, precisely [16]. β-lactamase enzymes help confer resistant bacterial phenotypes, as their mechanism of action hydrolyzes the β-lactam ring of such antibiotics, and the resulting chemical structure can no longer hinder bacterial cell wall synthesis [81]. These enzymes are so diverse that hundreds of them have been discovered and grouped into various classes in both Gram-negative and Gram-positive species [82]. Uniquely, in addition to β-lactamases, some *Staphylococcal* species contain the *oatA* gene that encodes an O-acetyltransferase enzyme, which is a major determinant allowing such species to avoid the inhibition of cell wall synthesis by lysozymes [83].

**Figure 4:**
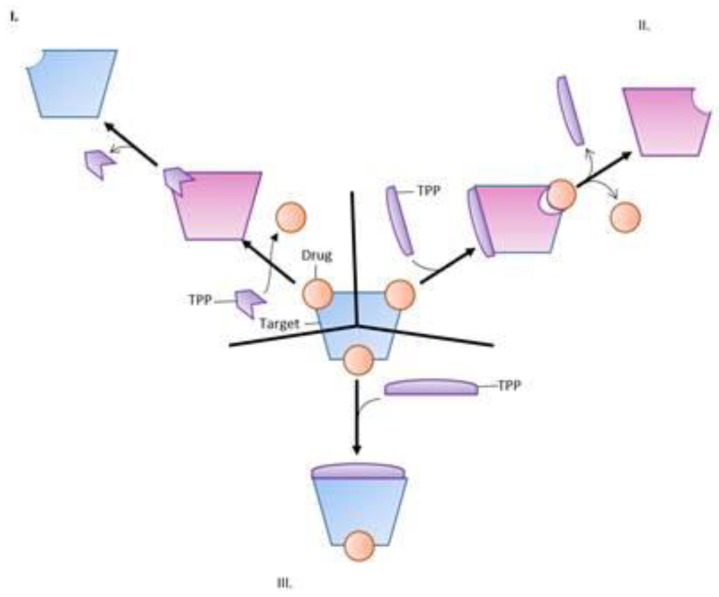
Types of antimicrobial target protection mechanisms. (**I**) The target protection protein (TPP) directly displaces the antimicrobial agent from its active site on the target, preventing antimicrobial action. (**II**) The target protection protein binds an allosteric site of the target, which induces a conformation change and the dissociation of the antimicrobial agent from the target site. (**III**) The target protection protein induces a global conformational change to reestablish target function despite the formation of a target-drug complex [84]. This figure kindly provided courtesy of Ann F. Varela.

In recent years, target protection has been a prominent mechanism for antimicrobial resistance in both Gram-positive and Gram-negative bacteria. There are no single and uniform target protection mechanisms. Three such mechanisms have been defined thus far, namely allosteric antibiotic removal, restoration of target function despite the presence of the bound antibiotic, and direct antibiotic displacement (see Figure 4) [84].

Some protein-encoding genetic determinants that mediate target protection have been found in bacterial chromosomes, and most of these determinants involved in this mechanism are carried out by mobile genetic elements [85]. For example, tetracycline (TetO and TetM), fusidic acid (FusB and FusC), and quinolone (Qnr) resistances occur through this mechanism.

### 4.1. Ribosomal Protection Protein (RPP)

Tetracycline ribosomal protection proteins facilitate target protection. To date, 13 classes of RPPs have been described, and the best-characterized RPPs are TetO and TetM [85]. TetM and TetO are soluble cytoplasmic proteins that were first isolated from *Streptococcus* spp. and *Campylobacter jejuni*, respectively, but genes coding these proteins are found in a wide range of bacteria [86,87]. These proteins belong to a translation factor superfamily of GTPases and act as a homolog to translation elongation factor G (EF-G) [88,89]. TetM and TetO mediate tetracycline resistance by interfering with the ability of the drug to bind to the ribosome [90]. They interact with the ribosome and catalyze the release of tetracycline from its binding site in a GTP dependent manner [91,92,93]. Structural studies have shown that both TetM and TetO overlap the tetracycline binding site on the ribosome, which indicates that the resistance of drugs is through direct displacement from the ribosome [88,94]. These RPPs alter the nucleotide conformation within a drug binding site and thus prevent the immediate rebinding of the drug and promote the delivery of the aminoacyl-tRNA [89].

### 4.2. Quinolone Resistance Proteins

The plasmid-mediated, quinolone resistance gene *qnr* is involved in quinolone and fluoroquinolone resistance in Gram-negative pathogens, such as in Enterobacteriaceae. This gene encodes pentapeptide repeat proteins, which mediate bacteria to resist quinolone inhibitory effects by binding and protecting the DNA gyrase and type II topoisomerases [5,95]. Several Qnr families (A, B, C, D, and S) have been identified to date, with QnrB having the most considerable number of alleles. Different studies showed that Qnr protein disrupts the DNA gyrase-quinolone interactions and increase quinolone efflux from the bacterial cell. The binding of Qnr to these enzymes decreases the affinity of the quinolone to stabilize with the complex that it forms with topoisomerase-cleaved DNA, thereby enabling the normal process and re-ligation of DNA [95].

## 5. Active Efflux Pumps of Antimicrobial Agents

In cases where intact antimicrobial agents enter bacterial cells and drug targets are freely accessible, active drug efflux systems can come into play. In this section, we will focus on well-studied antimicrobial transporters, as they make good model systems for study and resistance modulation. Bacteria that are pathogenic frequently make use of integral membrane proteins that function as transporters of antimicrobial agents [96]. Such bacterial transport proteins serve to actively export structurally distinctive antimicrobial agents from the cytoplasm, where drug targets reside, to the extracellular milieu, where their molecular targets are lacking [97]. Efflux pumps are present in all bacteria and are integral parts of bacterial physiology, being involved in diverse functions such as the expulsion of toxic products of metabolism, and maintenance of homeostasis. However, antibiotics as incidental substrates of efflux pumps have resulted in them being viewed largely as bacterial mechanisms of antimicrobial resistance. In clinically important bacteria, such as MDR *Mycobacterium tuberculosis*, methicillin-resistant *Staphylococcus aureus*, *Klebsiella pneumoniae,* and *Pseudomonas aeruginosa,* efflux pumps have critical roles in ensuring bacterial survival and evolution into resistant strains. These bacterial multidrug efflux pump systems are energetically driven by ATP hydrolysis, called primary active transport [98], and by electrochemical ion gradients or ion motive forces, called secondary active transport [99,100]. Active transport of antimicrobial agents represents an essential resistance mechanism in bacterial pathogens. As multiple structurally distinct antimicrobial agents with disparate modes of action are exported to the extracellular milieu, their growth inhibitory properties towards bacteria are diminished, if not wholly circumvented.

**Figure 5:**
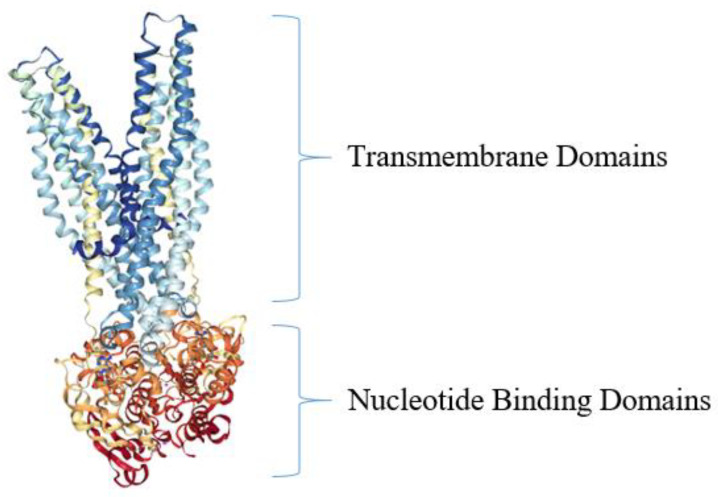
Crystal structure of bacterial ABC efflux pump from *S. aureus*. The top portion of the ABC transporter Sav1866 is depicted in blue and light blue and represents the two TMDs (sometimes called membrane-spanning domains, MSDs) of the protein, while the orange and red colors depict the two NBDs [104]. The model structure was generated using NGL Viewer [106] of the PDB [107] entries 2HYD and 2ONJ, as reported [104,108].

During the primary active transport of antimicrobial agents, bacteria exploit the biological energy stored in the form of intact adenosine triphosphate (ATP) to export drugs against the drug concentration gradient by performing ATP hydrolysis [25]. During the export of antibacterial agents from bacterial cells, ATP is hydrolyzed in order to energize the drug translocation through the transporter in an outward direction across the membrane. Thus, as the transporter substrate actively accumulates outside the cell, drug resistance is conferred upon the bacterial pathogen [98]. One of the best-studied of these primary active drug efflux systems in bacteria is the ATP-binding cassette (ABC) efflux pump family [101,102]. The ABC transporter superfamily represents one of the most abundant protein families known across all taxa of living organisms [103]. One of the first of the bacterial ABC efflux pump structures to be determined was Sav1866, from the pathogen *S. aureus* [104] (Figure 5). Structurally speaking, the Sav1866 drug efflux pumps consist of two chief transmembrane domains (TMDs) and two nucleotide-binding domains (NBDs) [104]. During translocation and efflux of the antimicrobial agent across the bacterial membrane, a conformational change occurs in the TMDs in order to accommodate substrate binding and transport [105]. Meanwhile, as the antimicrobial agent is pumped to the outside of *S. aureus* cells, ATP is hydrolyzed to adenosine diphosphate (ADP) in the interior of the cell by the NBDs, which harbor ATPase activities [104,105].

The ABC group of efflux pumps prompt bacterial recalcitrance to clinically relevant drugs in *Mycobacterium tuberculosis*, *Acinetobacter baumannii*, *Streptococcus pneumoniae*, *Staphylococcus aureus*, etc. MsrA, widely distributed in Gram-positive and -negative organisms, is responsible for macrolide resistance [109]. An erythromycin inducible MsrA homolog efflux pump, Mel, mediates macrolide resistance in *Streptococcus pneumoniae* together with MefE [110]. Higher expression of ABC efflux pumps Rv1217c, and Rv1218c resulted in increased MIC of rifampicin, while the overexpression of Rv1218c increased the MIC of isoniazid [111]. In *S. pneumoniae*, the ABC efflux pumps PatA and PatB confer resistance to clinically relevant drugs such as the fluoroquinolones and are overexpressed in clinical isolates [112].

The MacB efflux pump of *E. coli* is one of the few well-studied efflux proteins of the ABC superfamily, which confers appreciable levels of resistance to macrolides [113]. This protein, together with its outer membrane protein MacA, has been shown to have a crucial role in the virulence of *E. coli*. In *Salmonella* Typhimurium, MacABC is necessary for host colonization, and it helps the bacterium to overcome the lethal oxidative stress induced by the reactive oxygen species (ROS) and aids in its survival inside macrophages [114].

**Figure 6:**
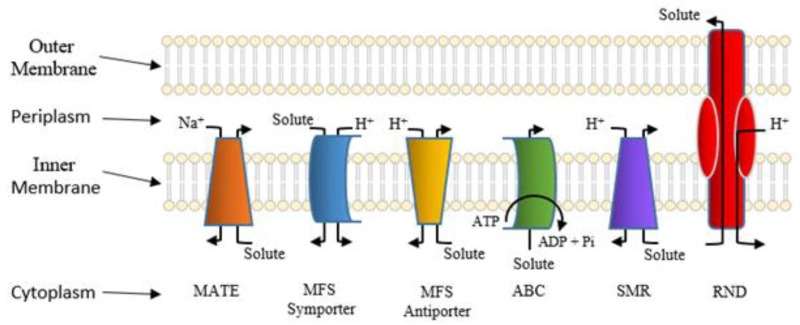
Classes of well-studied bacterial solute transporters. The bacterial outer and inner (cytoplasmic) membranes are shown. Also depicted are the cytoplasmic and periplasmic spaces. P_i_ denotes phosphate, and Na^+^ and H^+^ denote sodium and proton, respectively. This figure kindly provided courtesy of Ann F. Varela.

Secondary active transporters also confer bacterial resistance to many structurally distinctive antimicrobial agents [115,116]. Throughout the last 30 years, these active antimicrobial efflux pump systems have been categorized into several large superfamilies of related proteins based on similarities in amino acid sequences, structures, and modes of energization [117,118]. Currently, these superfamilies are denoted as follows: the major facilitator superfamily (MFS) [119]; the drug/metabolite transporter (DMT) superfamily, which now harbors the small multidrug resistance (SMR) family [120,121]; the multidrug and toxic compound extrusion (MATE) family, which has been included within the larger multidrug/oligosaccharidyl-lipid/polysaccharide (MOP) superfamily of transporters [122,123]; the proteobacterial antimicrobial compound efflux (PACE) transporter superfamily [124]; and the resistance-nodulation-cell division (RND) superfamily [125]. Several well-studied families of bacterial solute transporter systems are shown in Figure 6.

**Figure 7:**
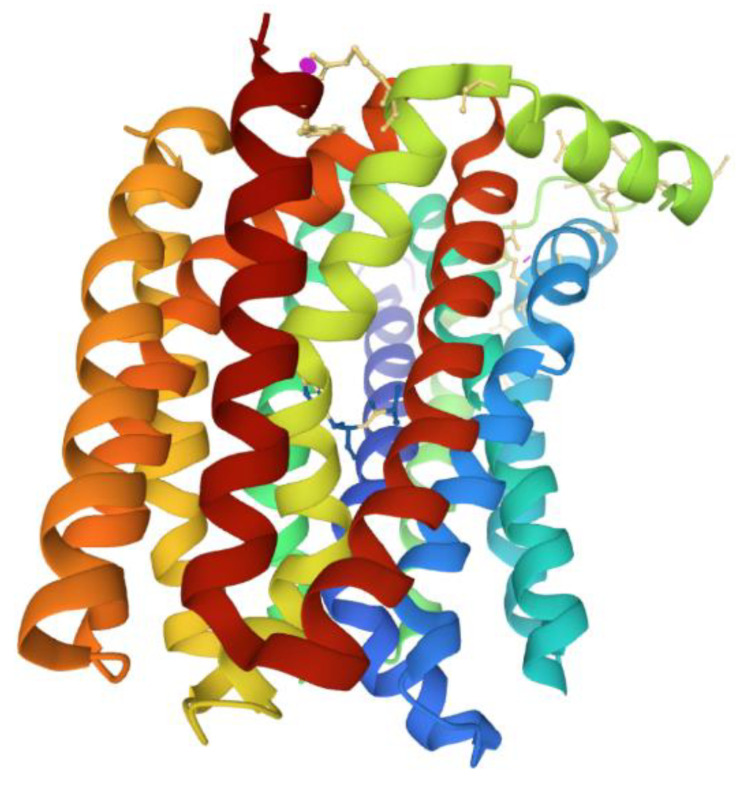
Crystal structure of *E. coli* MdfA multidrug efflux pump from the MFS. The MdfA transporter is complexed to one of its substrates, chloramphenicol (ball and stick structure). Ribbons of different colors represent the transmembrane helices. The loops between the transmembrane domains were removed for clarity. The model of the MdfA structure was generated using NGL Viewer [106] from the Protein Database, PDB [107], entry 4ZOW from Heng et al. [130].

Many members of the MFS of bacterial efflux pumps confer resistance to multiple antimicrobial agents and are considered essential molecular targets for resistance modulation in order to circumvent resistance and restore the therapeutic efficacy of compromised agents [126,127]. The protein structures for several bacterial antimicrobial efflux pumps belonging to the MFS have been elucidated [128]. In general, the MFS structures harbor 12 or 14 α-helical transmembrane segments, two seemingly symmetrical bundles, each belonging to either the N- or C-terminal ends, the so-called MFS fold consisting of adjacent triplet α-helices, and functional highly conserved amino acid sequence motifs [128,129]. Recently, protein structure studies of the MdfA multidrug efflux pump from *E. coli* showed bound substrates, such as chloramphenicol [130] (Figure 7), and inhibitors, [130,131], plus a crystal structure composed of a periplasmic-facing conformation suggesting a functional role for the highly conserved antiporter motif C sequence in conducting substrate translocation through the antimicrobial pumps [132,133,134]. Studies like these will undoubtedly play crucial roles in the evaluation of the physiological mechanisms for antimicrobial efflux across the membrane and their exploitation for the development of efflux pump inhibition [135].

Some of the clinically relevant and intensely studied MFS efflux pumps belong to *Staphylococcus aureus*, including NorA, NorB, NorC, QacA, QacB, TetA(K), LmrS, and MsrA [136]. These efflux pumps directly or indirectly contribute to the ability of *Staphylococcus aureus* to tolerate antibiotics, such as by decreasing intracellular concentration of antibiotics, which allows bacteria to survive longer in the presence of antibiotics and develop resistance through other mechanisms involving gene mutations, overexpression of porins, etc. In *S. aureus*, the NorA efflux pump promotes the development of ciprofloxacin resistance directly or by positively contributing to the fitness advantage provided by topoisomerase gene mutations [137]. The elevated levels of *norA* expression potentiate ciprofloxacin resistance, although this phenomenon is highly variable across clinical staphylococcal strains [137]. Inhibition of the NorA efflux pump with a clinically approved drug nilotinib diminished the biofilm formation by *S. aureus*, and this drug can potentiate ciprofloxacin activity in clinical settings [138]. Obviously, efflux pumps are key components of complex circuits involving antibiotic resistance, persistence, and virulence [139].

With the discovery of the SMR family and its subsequent incorporation into the larger DMT superfamily arose the elucidation of a low-resolution crystal structure for the DMT-based antimicrobial efflux pump, called EmrE, which has been an effective model system for antimicrobial transport [121,140,141]. While the structural nature of EmrE has been controversial in terms of the monomer orientation for its dimer [141,142], molecular dynamics simulations, biochemical, and physiological studies pertaining to the structure-function relationships and efflux inhibition have shed new light on its substrate translocation mechanism [143,144,145,146,147].

The crystal structure of the RND transporter AcrB from *E. coli*, first reported in 2002 [148], consists of a trimer [149,150]. The AcrB trimer component is known to reside within the inner membrane of Gram-negative bacteria [151]. In one mechanistic model for antimicrobial transport, the AcrB is thought to rotate in a manner akin to a peristaltic pump in which the pump repeatedly cycles between extrusion, access, and binding steps [152,153]. Furthermore, the AcrB efflux pump has been demonstrated to assemble into a tripartite multi-complex assembly with a periplasmic-located protein, AcrA, and an outer-membrane protein, TolC [154]. This tripartite antimicrobial drug efflux system has been found in a variety of life-threatening bacterial pathogens and confers resistance to multiple clinically relevant antibacterial agents [155]

The bacterial RND tripartite multidrug efflux pump systems from *E. coli* consists of three main domains constituting a tripartite structure. The top third of the structure denotes the outer membrane-associated channel, TolC; the middle section includes the periplasmic-associated domain, AcrA, and the third section is constituted by AcrB, an extensively studied member of the RND superfamily [150].

In general, these distinctive families of antimicrobial transporter systems serve to confer bacterial pathogens enhanced capabilities to survive antimicrobial stress [136]. Apart from AcrB-TolC, some of the extensively studied, clinically relevant RND efflux pumps are MexB, MexF, and MexY of *Pseudomonas aeruginosa*, AdeB of *Acinetobacter baumannii*, CmeB of *Campylobacter jejuni*, and MtrD of *Neisseria gonorrhoeae* in Gram-negative bacteria [156]. In *Bacteroides fragilis* clinical isolates, *bmeB* efflux pump overexpression coupled with GyrA point mutations contribute to a clinical level of resistance to fluoroquinolone and β-lactams [157]. A recent study suggests that the AcrAB efflux pump has a role in the initial stages of bacterial transition from transient antibiotic resistance to permanent resistance. The lower expression of DNA repair gene *mutS* in *acrAB* overexpressing strains contributes to higher frequencies of spontaneous mutations and hence higher probabilities of resistance development [158]. Therefore, the presence of an efflux pump and its expression level cannot be viewed in isolation but should be correlated with other mechanisms of resistance that might act in synergy with efflux pumps. Consequently, these drug transport systems represent desirable targets for inhibitors [159] in order to circumvent resistance and restore the therapeutic efficacy of multidrug-resistant bacterial pathogens [10,126,127,128,136,160]. Therefore, molecular studies of transporter structures and efflux mechanisms will undoubtedly continue to be relevant in the foreseeable future [161].

Unfortunately, fundamental knowledge of the molecular mechanisms for multidrug transport is lacking. For example, we still know little about the modes for tying together energetic systems versus antimicrobial translocation across the membrane. Further, we do not yet understand how antimicrobial transporters dictate multiple substrate transport while preventing the passage of unwanted substrates or leakages of relatively smaller ions, like sodium ions or protons. For many if not all of these antimicrobial transporters we do not yet have a clear picture of the nature of the structural configurations assumed during each of the specific steps of their transport cycles. In summary, much work remains to be performed before we can clearly understand the physiology of antimicrobial transport both at fundamental and applied levels of investigation.

## 6. Reduction of Antimicrobial Permeability into Bacterial Cells

In contrast to active drug efflux systems where export is an effective means of resistance, bacterial may also simply prevent the entry of antimicrobial agents. An important mechanism of bacterial resistance to antimicrobial agents involves preventing drug permeability and access to the internal milieu of the pathogenic cells [162]. Strains of Gram-negative pathogenic bacterial species, such as *Escherichia coli*, *Pseudomonas aeruginosa*, *Vibrio cholerae*, *Klebsiella* spp., and *Salmonella enterica*, are particularly troublesome [163]. The molecular systems involved in reduced permeability of antimicrobial agents include resistance mechanisms at the bacterial cell wall. The extensive structural nature of the lipopolysaccharide layer constitutes a formidable barrier to the passage of small molecules, especially those that are growth inhibitory in their properties [164]. Another important molecular mechanism for conferring resistance via permeability reduction involves porins, which are integral outer membrane proteins with water-filled pore-like channels that permit the passage of molecules with definitive sizes and charges [165]. The relationship between bacterial antimicrobial resistance and the outer membrane porins can take one of several avenues. A wide-type porin can be highly selective towards the entry of certain nutrients, like sugars, and not permit the passage of many antimicrobial agents [165]. However, for those porins for which no such highly selective properties are a problem, then in such cases, the porin molecules may be depleted from the membrane or functionally disrupted by mutation [165,166]. In other cases, permissive porins may be regulated by channel blockers or by RNA-specific antisense modulators [167,168].

**Figure 8:**
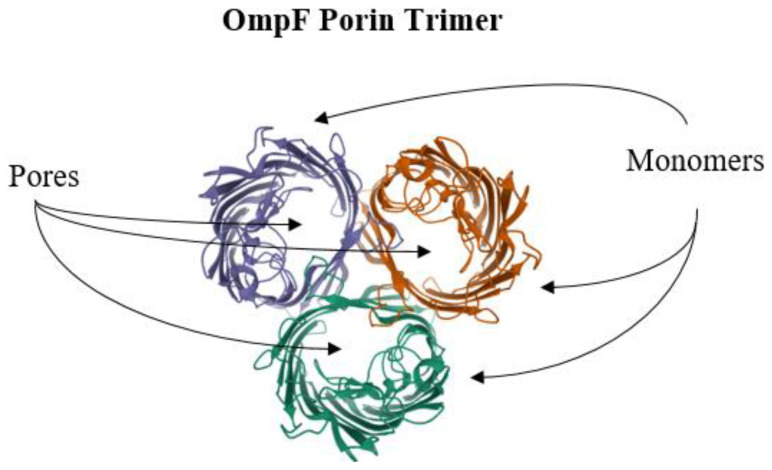
Outer membrane protein, OmpF, is a porin from *Escherichia coli*. The OmpF porin is a trimeric apparatus consisting of three monomers. The OmpF porin structure was generated with the NGL Viewer [106] from the Protein Database, PDB [107], entry 2OMF from Cowan et al. [174].

One well-known antimicrobial resistance-conferring porin system is that of the *E. coli* and its outer membrane proteins OmpC, OmpF, and PhoE [149,151]. Other well-studied porin systems are from *Acinetobacter baumannii* and OprD from *P. aeruginosa*, both microorganisms recognized as severe pathogens [169,170]. The crystal structures for porins have been solved to high resolution. For example, the OmpF structure from *E. coli* was one of the earliest and best understood of the porins (Figure 8) [171,172], and the OprO structure from *P. aeruginosa* is one of the most recent examples for which high-resolution porin molecules have been determined [173].

The overall OmpF porin structure consists of three monomers to constitute a trimeric apparatus (Figure 8) [174]. Each of the monomers is composed of β-stranded transmembrane elements to produce a gated β-barrel structural motif [175]. Molecular physiological data suggest that each of the monomers harbor binding sites for antimicrobial agents [165]. The degree of the selective natures for substrates is an ongoing focus of investigative studies, and much molecular work remains to be conducted in order to definitively demonstrate the precise molecular pathways of water-soluble substrates through their dedicated channels, as well as their gating mechanisms.

The lipopolysaccharide (LPS) layer of Gram-negative bacteria plays a very important and direct role in antibiotic resistance. Being the outermost layer of Gram-negative bacteria, LPS comes in direct contact with antibacterial compounds, and its interactions with them decide the susceptibility of bacteria to inhibitory compounds. Cationic antimicrobial peptides (CAMPs) are a group of antibacterials that specifically interact with LPS and disrupt it by displacing divalent cations, that stabilize LPS by neutralizing its negative charge [176]. In *E. coli*, the composition of the core oligosaccharide of LPS and the sugar composition of the outer part determine the susceptibility to antimicrobial peptides [177]. LPS is also a target for peptide antibiotics colistin and polymyxin B. These cationic peptide antibiotics interact with the phosphoric acid group of lipid A and replace calcium and magnesium ions associated with it leading to destabilization of LPS and leakage of cellular contents that eventually causes the death of bacteria [178]. Resistance to the peptide antibiotic colistin mediated by *mcr* genes involves *hpap2* or *dgkA* genes that encode putative phosphatidic acid phosphatase of type 2 (PAP2) and diacylglycerol kinase, respectively which are involved in LPS biosynthesis [179]. In *E. coli,* a complex interaction of proteins, such as PmrA, PmrD, and PhoPQ, is involved in modifying lipid A under Mg^2+^-limiting growth conditions, eventually leading to bacterial resistance to cationic antimicrobial peptides such as polymyxin B [180]. A distinctly different mechanism of antibacterial action is exhibited by cationic antimicrobial peptide thanatin that acts on bacteria by cell agglutination upon interacting with LPS [181]. This peptide is effective against diverse multidrug-resistant Gram-negative bacteria [182]. However, the mechanisms of inhibitory activities of thanatin against Gram-positive bacteria and fungi are not clearly elucidated. AMPs such as thanatin have revived the hope of developing effecting antimicrobial therapies, either alone or in combination with antibiotics, against extremely drug-resistant bacteria [183].

## 7. Future Directions

Bacterial pathogens are critically essential causative agents of severe infectious disease [184]. As such, much effort has gone into the development of chemotherapy in addressing high morbidity and mortality numbers [185,186]. Therefore, continued investigation towards the improvements in personal hygiene methods, food handling and preparation, hand washing, public sanitation, and education across all levels will be the focus of intense interest.

In medical healthcare and treatment centers, antimicrobial stewardship is still a promising approach, and much effort continues to be centered towards further development [187,188]. Attention will undoubtedly need to be paid towards studies of multidrug resistance in bacteria found in veterinary medicine and agricultural practices to reduce infection transmission and persistence in these areas [9].

New incentives to discover new antibacterial agents with novel modes of action are few, and progress on this front is slow [189,190]. A promising avenue in the battle against multidrug-resistant pathogens entails the clinical investigation of non-antibiotic agents as anti-bacterial agents, such as non-steroidal anti-inflammatory agents, anesthetics, and statins [191]. Recently, a series of new and well-developed anti-infective strategies for the circumvention of multidrug-resistant pathogens were reviewed elsewhere [10]. These and other strategic modes for reducing the conditions that foster the spread of bacterial infections are prime candidates for enhanced efforts of investigation.

## 8. Concluding Remarks

Bacterial pathogens that have acquired specific antimicrobial resistance mechanisms have emerged as serious clinical agents of infection, causing a public health concern on a worldwide scale. Such cellular mechanisms of antimicrobial resistance include multidrug efflux pumps, enzymatic drug degradation, biofilm formation, drug target modification, and target protection. Many genetic determinants for bacterial antimicrobial resistance are transferable to unrelated species, having evolved new means of movement through human populations. To reduce the conditions that foster the emergence and spread of clinical infections new strategies have been considered. Future directions include the development of new chemotherapeutics, such as those with novel cellular targets, the continuation of public health practices, education, clinical antimicrobial stewardship, and continued molecular investigation of resistance mechanisms.

## Abbreviations

ABC	ATP-binding cassette
AAC	*N*-acetyltransferases
AME	aminoglycoside-modifying enzymes
ANT	O-adenyltransferases
APH	O-phosphotransferases
CAT	chloramphenicol acetyltransferase
CRE	carbapenem-resistant Enterobacteriaceae
CTX-M	cefotaximase
ESBLs	extended-spectrum β-lactamases
ESKAPE	*Enterococcus, S. aureus, K. pneumoniae, A. baumannii, P. aeruginosa, and E. coli*
MATE	multidrug and toxic compound extrusion
MFS	major facilitator superfamily
MOP	multidrug/oligosaccharidyl-lipid/polysaccharide
NAG	*N*-acetylglucosamine
NAM	*N*-acetylmuramic acid
NBDs	nucleotide-binding domains
NDM	New Delhi Metallo-β-lactamase
PACE	proteobacterial antimicrobial compound efflux
PBPs	penicillin-binding proteins
RND	resistance-nodulation-cell division
TEM	temoneira
TMDs	transmembrane domains
SHV	sulfhydryl variable
